# Pattern of Neurogenesis and Identification of Neuronal Progenitor Subtypes during Pallial Development in *Xenopus laevis*

**DOI:** 10.3389/fnana.2017.00024

**Published:** 2017-03-27

**Authors:** Nerea Moreno, Agustín González

**Affiliations:** Department of Cell Biology, Faculty of Biology, Complutense University of MadridMadrid, Spain

**Keywords:** telencephalon, proliferation, radial glial cells, intermediate progenitors, Pax6, Tbr2, evolution

## Abstract

The complexity of the pallium during evolution has increased dramatically in many different respects. The highest level of complexity is found in mammals, where most of the pallium (cortex) shows a layered organization and neurons are generated during development following an inside-out order, a sequence not observed in other amniotes (birds and reptiles). Species-differences may be related to major neurogenetic events, from the neural progenitors that divide and produce all pallial cells. In mammals, two main types of precursors have been described, primary precursor cells in the ventricular zone (vz; also called radial glial cells or apical progenitors) and secondary precursor cells (called basal or intermediate progenitors) separated from the ventricle surface. Previous studies suggested that pallial neurogenetic cells, and especially the intermediate progenitors, evolved independently in mammalian and sauropsid lineages. In the present study, we examined pallial neurogenesis in the amphibian *Xenopus laevis*, a representative species of the only group of tetrapods that are anamniotes. The pattern of pallial proliferation during embryonic and larval development was studied, together with a multiple immunohistochemical analysis of putative progenitor cells. We found that there are two phases of progenitor divisions in the developing pallium that, following the radial unit concept from the ventricle to the mantle, finally result in an outside-in order of mature neurons, what seems to be the primitive condition of vertebrates. Gene expressions of key transcription factors that characterize radial glial cells in the vz were demonstrated in *Xenopus*. In addition, although mitotic cells were corroborated outside the vz, the expression pattern of markers for intermediate progenitors differed from mammals.

## Introduction

The pallium constitutes the most complex region of the vertebrate brain, especially due to the organization and functionality of its derivatives, which makes it the most sophisticated and elaborate structure, in relation to the achievement of higher cognitive abilities that characterize humans. Current research on the organization of the pallium of different vertebrates in search for the origin of its organization aims to solve one of the most urgent and crucial issues of recent years in the evolutionary-developmental (evo-devo) approach. In particular, we can get a deeper understanding of the development of the pallium by studying this process in different model vertebrates and trying to unravel the evolutionary changes that can be inferred (Nomura et al., 2016).

The complexity of the pallium during evolution has increased dramatically in many different respects. The highest level of complexity is found in mammals, where the pallium (cortex) mainly shows a layered organization, with only minor nuclei formation. In the cortex, functional areas organized into columns can be recognized, and most cells that belong to the same area or column in each layer are generated within the same sector of the vz and, subsequently, migrate radially toward the mantle area, which has given rise to the concept of “radial unit” (reviewed in Rakic, 2007), with an inside-out formation sequence (later-born neurons migrate beyond the cells located in deep older layers to end in a more superficial position; reviewed in Medina and Abellán (2009). However, in non-mammalian amniotes (birds and reptiles), the inside-out sequence of layer organization is not present, and the pallial cells are generated in an opposite outside-in order (the oldest cells are located closer to the surface). In addition, in the most complex of cases, a three-layered pallium is formed, far from the sophisticated structure of mammals (Charvet et al., 2009). Within this complex scenario, recently some authors have postulated that the various subtypes of neurons that are layer specific in mammals do exist in the chicken pallium, and the neurons of deep and upper layers are segregated into different mediolateral domains (Suzuki and Hirata, 2011, 2013, 2014; Suzuki et al., 2012). However, there is great controversy over this proposal (Medina et al., 2013; Puelles et al., 2016).

In the search for the ontogenetic mechanisms underlying the expansion of the neocortex, many recent studies have shed light on the differences between species in major neurogenetic events (Clinton et al., 2014; Martínez-Cerdeño et al., 2016). Pallial neurogenesis is understood as the process of generation of post-mitotic cells, neurons and glia, from the neural progenitors that divide and produce all the cells of the pallium. This process is found in all vertebrates and although there are phylotypic differences, it always begins very early in development and continues in later stages, and even in the adult. In general terms, in the case of mammals, authors have described primary precursor cells, also called radial glial cells (RGc) or apical progenitors, which are Pax6+ bipolar dividing cells at the ventricular surface, and secondary precursor cells, called basal or intermediate progenitors (IPs), which are Tbr2+ multipolar dividing cells separated from the ventricular surface (reviewed in Martínez-Cerdeño and Noctor, 2016; Montiel et al., 2016). In an evolutionary context, previous studies have suggested that pallial neurogenetic cells, and especially the IPs, evolved independently in mammalian and sauropsid lineages. For example, in some reptiles such as crocodiles or geckos, no IPs were detected (Charvet et al., 2009; Martínez-Cerdeño et al., 2016), but they were described in turtles (Cheung et al., 2007; Clinton et al., 2014; Martínez-Cerdeño et al., 2016). Therefore, this feature opens a very interesting question about evolutionary relationships, because the presence of IPs may be a derived feature of the turtle lineage that evolved independently, or they may had been already present in ancestral amniotes or even in anamniotes. Interestingly, the striking expansion of the cortex in development and evolution has been related to the appearance of more abundant IPs with high capacity of proliferation (reviewed in Namba and Huttner, 2016).

In this context, it is worth noting that recent studies of the early neurogenesis in anamniotes (commonly and incorrectly called lower vertebrates in many texts) has constituted an important motor for the development of this field of research, since some of the main advances were first described in species such as *Xenopus* or zebrafish (Piccolo et al., 1996; Zimmerman et al., 1996; Adolf et al., 2006; März et al., 2010; Rothenaigner et al., 2011). But in addition, from an evolutionary point of view species like *Xenopus* provide key clues because amphibians represent the only anamniote tetrapods that accomplish development through embryonic to larval and juvenile stages, with a metamorphic process in which the neurogenic capabilities vary, thus allowing a very interesting scenario for this type of analysis. In the present study, we examined the main pallial features in terms of neurogenesis from the pallial progenitors of the *Xenopus laevis* telencephalon, from embryonic to juvenile stages. We have analyzed how neural progenitors proliferate and the cell-birth rate by BrdU assays throughout the course of embryonic, larval and post-metamorphic development. BrdU is a specific marker for the S-phase, and the incorporation of BrdU into the DNA serves for the identification of newborn cells (reviewed in von Bohlen und Halbach, 2011). Additionally, we have used the markers phosphorylated form of histone H3 (phosphohistone H3; PH3) and proliferating cell nuclear antigen (PCNA) for cell proliferation identification. PH3 is a component of the histone octamer, which is present in the cell division along the late G2 phase and in the M phase (Hendzel et al., 1997), whereas PCNA is a DNA polymerase-delta subunit involved in DNA replication and error repair (Zacchetti et al., 2003). It is highly expressed along G1 and S-phases, whereas in G2 and M-phases its expression is reduced. The localization of these markers has been analyzed immunohistochemically in combination with the detection of other markers such as the brain lipid-binding protein (BLBP, a marker of the RGc during brain development and in the adult; Pinto and Götz, 2007), SRY-related HMG-box gene2 (Sox2, a marker of neural stem and progenitor cells; Kamachi and Kondoh, 2013; Sarkar and Hochedlinger, 2013), and doublecortin (DCX, protein expressed in neuroblasts during migration and in young neurons; von Bohlen und Halbach, 2011). Pax6 and Lhx2, have been used to label pallial precursors, because throughout cortical neurogenesis they have been described as markers of neocortical progenitors within the vz, and both are involved in cortical cell fate determination (reviewed in von Bohlen und Halbach, 2011; Chou and O’Leary, 2013). Finally, the staining with Tbr2 as marker of IPs was attempted (Noctor et al., 2004; Martínez-Cerdeño et al., 2016).

Our results show that the mitotic rate increases from embryonic stages of development to early larvae, when the animal has a period of quiescence until mid larval stages, when a neurogenic peak is reached, which later gradually decreases until the juvenile frogletts stages. Accordingly, there are two waves of progenitor divisions, one at the mid embryonic period and other at mid larval development. The sequence of pallial development follows an outside-in order, and the differentiating cells are accumulated to the mantle, following the concept of radial unity. Pax6 and Lhx2 are early expressed in the ventricular proliferative zone and later in postmitotic cells separated from the ventricle, whereas Sox2 mitotic cells are present in ventricular and abventricular zones, and some of those cells express DCX. Finally, Tbr2 is not expressed in mitotic abventricular cells.

## Materials and Methods

### Animals

For the present study embryonic and larval specimens of the African clawed frog *Xenopus laevis* were used. They were sorted by stages following Nieuwkoop and Faber (1967) and grouped into embryonic (35–45), premetamorphic (46–52), prometamorphic (53–58), and metamorphic (59–65) stages (**Table 1**). The regulations and laws of the European Union (2010/63/EU) and Spain (Royal Decree 53/2013) were strictly followed for the care and handling of the animals in our research, and the experiments designed for this study were approved by the Complutense University. Adult males and females were commercially purchased from the CNRS colony (Montpellier or Rennes, France). *In vitro* fertilization, after human chorionic gonadotropin (HCG)-induced egg-laying, was carried out to obtain the different developmental stages. The animals were kept in tap water at 20–25°C and after reaching the appropriate embryonic or larval stages, they were anesthetized by immersion in a 0.3% solution of tricaine methanesulfonate (MS222, pH 7.4; Sigma–Aldrich, Steinheim, Germany).

**Table 1:** *Xenopus laevis* developmental timing at 23°C.

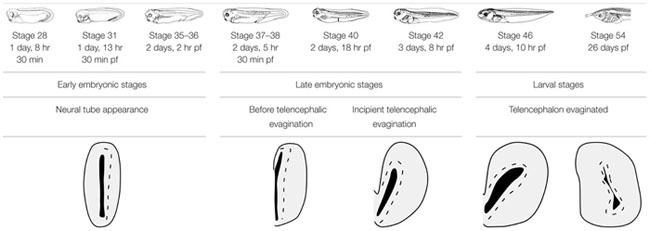

### Immunohistochemistry

Single and combined immunohistofluorescence, two-step protocols were conducted with cocktails of the primary antibodies listed in **Table 2**. For fixation, embryos and premetamorphic larvae were fixed by immersion overnight at 4°C in MEMFA (0.1 M MOPS [4-morpholinopropanesulphonic acid], 2 mM ethylene glycol tetraacetic acid, 1 mM MgSO4, 3.7% formaldehyde). The brains were dissected out and then processed in toto. Subsequently, they were immersed in a solution of 30% sucrose a 0.1 M phosphate buffer (PB; pH 7.4) for 5 h at 4°C until they sank, embedded in a solution of 20% gelatin with 30% sucrose in PB, and then immersed in a 3.7% formaldehyde solution at 4°C for 14–20 h. By means of a freezing microtome (Thermo Scientific Microm HM 450) the brains were cut at 18–30 μm in the transverse plane and the sections were collected and rinsed in cold PB. Larger animals, at the final larval stages (from stage 54), were perfused transcardially with 0.9% NaCl solution, followed by 10–100 ml of MEMFA. The brains were removed, blocked in gelatin (as described above) and cut on a freezing microtome at 18–30 μm in the transverse, horizontal, or sagittal plane (see Domínguez et al., 2013).

**Table 2:** Antibodies used in the present study.

Name	Immunogen	Commercial supplier	Dilution
BLBP	Synthetic peptide conjugated to KLH derived from within residues 1 – 100 of Mouse BLBP.	Polyclonal rabbit anti-BLBP. Abcam. Catalog reference: ab32423	1:500
BrdU	BrdU	Monoclonal mouse anti-BrdU. Developmental Studies Hybridoma Bank. Catalog reference: G3G4	1:500
CR	Recombinant human calretinin	Polyclonal rabbit anti-CR. Swant. Catalog reference: 7699/3H	1:1,000
DCX	Epitope mapping at the C-terminus of Doublecortin of human origin	Polyclonal goat anti-DCX. Santa Cruz. Catalog reference: sc-8066	1:500
GABA	*c*-Aminobutyric acid (GABA) conjugated to BSA	Polyclonal rabbit anti-c aminobutyric acid. Sigma, St. Louis MO, USA. Catalog reference: A2052	1:3000
Islet 1	Amino acids 247–349 at the C-terminus of rat Isl1	Monoclonal mouse anti-Isl 1. Developmental Studies Hybridoma Bank. Catalog reference: 39.4D5	1 :500
Lhx2	Amino acids: 231–406	Monoclonal mouse anti-Lhx2. Developmental Studies Hybridoma Bank. Catalog reference: 2C10	1:50
PAX6	Peptide sequence: QVPGSEPDMSQYWPRLQ of the C-terminus of mouse PAX6 protein	Polyclonal rabbit anti-PAX6. Covance. Catalog reference: PBR-278	1:250
PCNA	Protein A-rat PCNA (proliferating cell nuclear antigen) fusion obtained from pC2T	Monoclonal mouse anti-PCNA. Abcam. Catalog reference: PC10	1:500
PH3	Amino acid sequence containing phosphorylated Ser 10 of Histone H3	Polyclonal rabbit anti- p-Histone H3 (Ser 10)-R. Santa Cruz. Catalog reference: sc-8656-R	1:500
Sox2	Synthetic peptide conjugated to KLH derived from within residues 300 to the C-terminus of Human SOX2.	Polyclonal rabbit anti-Sox2. Abcam. Catalog reference: ab97959	1:500
Tbr1	Amino acids 1-200 at the N-terminus of mouse TBR-1	Polyclonal rabbit anti-Tbr-1. Santa Cruz Biotechnology. Catalog reference: sc-48816	1:1000
Tbr2	Peptide mapping at the N-terminus of Eomes of human origin	Polyclonal goat anti-Tbr-2. Santa Cruz Biotechnology. Catalog reference: sc-69269	1:500

Protocols for immunohistofluorescence were carried out in the free-floating sections, or in toto for the cases of embryos and young larvae. The steps in the procedure were as follows: (1) First incubation, conducted for 48 h at 4°C in the dilution of each primary antibody (see **Table 1**). For the double labeling of two different markers in the same section, cocktails of pairs of primary antibodies developed in different species were used at the same dilutions and conditions specified. (2) According to the species in which the primary antibody was raised, second incubations were conducted with the appropriately labeled secondary antibody diluted 1:500 for 90 min at room temperature: Alexa 594-conjugated goat anti-rabbit (Molecular Probes, Eugene, OR, USA; catalog reference A11037) and Alexa 488-conjugated goat anti-mouse (Molecular Probes; catalog reference A21042), or Alexa 594-conjugated donkey anti-goat (Molecular Probes, Eugene, OR, USA; catalog reference A-11058) and Alexa 488-conjugated chicken anti-rabbit (Molecular Probes; catalog reference A-21441), or Alexa 594-conjugated donkey anti-goat (Molecular Probes, Eugene, OR, USA; catalog reference A-11058) and Alexa 488-conjugated chicken anti-mouse (Molecular Probes; catalog reference: A-21200). In all cases the antibodies were diluted in PB containing 0.5–1% Triton X-100. After being rinsed, the sections were coverslipped with fluorescence mounting medium, containing 1.5 μg/ml 4′,6-diamidino-2-phenylindole (DAPI) for DNA counterstaining (Santa Cruz; SC-24941 or Vectashield H-1500).

### Controls and Specificity of the Antibodies

The commercial suppliers of the different antibodies used assessed their specificity (**Table 1**). In addition, controls for the immunohistochemical procedures were performed as in our previous studies on amphibians. The controls included: (1) the omission of the primary or secondary antibody in the incubation solution, (2) the use of preimmune mouse or rabbit sera in substituon of the primary antibody, and/or 3) Western blot analysis (Moreno et al., 2008a,b; Moreno et al., 2012; Morona and González, 2008; Domínguez et al., 2013).

### Quantification of PH3-Immunoreactive Cells

The antibody used to localize PH3 is suitable for the detection of mitotic cells, as it has been demonstrated in several species, including *Xenopus*, where the immunolabeling obtained was found consistently in mitotically active nuclei primarily located in the ventricular lining (McKeown et al., 2013). We observed that this antibody labels cell nuclei that display morphological features characteristic of various phases of mitosis. For the quantification of PH3 immunoreactive (PH3-ir) cells all immunopositive cells of the pallium were counted, in sections at specimens at stages 37/38, 46 and 54. Cells were systematically counted in alternate serial sections in order to avoid double cell counting. The number of sections in each serial set depended on pallial size at each developmental stage. Additionally, the sections were processed for Isl1 immunohistochemistry for the identification of the pallial/subpallial boundary. The surface of each section was measured on digital microphotographs using ImageJ free software^1^. The pallial rate of mitosis was expressed as the number of PH3-ir cells/area. Additionally, the cells were counted separately in the pallium and the SPa and in two different pallial zones (medial/dorsal and lateral/ventral) in alternate serial sections of *X. laevis* at stages 37/38, 42, 46 and 54. Statistical analyses were performed using Statgraphics software (Statgraphics centurion XVII). We confirmed the normal distribution of the groups of data by the Kolmogorov–Smirnov test. Variance of samples was checked by *F*-test. The groups of data were compared by one-way ANOVA followed by a *post hoc* Bonferroni test. In three cases the data were log10 transformed prior to ANOVA (pallium stage 46 versus stage 54; pallium versus SPa stage 42; DM versus VL stage 37/38) and in these cases the data were additionally analyzed by Kruskal–Wallis test, with comparable results of significance.

### BrdU Labeling and Tissue Processing

Two types of approaches were used: (1) for neural birth dating BrdU (Sigma B5002) was administered to stages 28, 31 and 37/38 (embryonic stages before the pallial telencephalic evagination; see **Table 1**) by immersion for 30 min at room temperature in 20 mM BrdU in 0,1X Marc’s Modified Ringers (MMR: 1 M NaCl; 20 mM KCl; 10 mM MgCl2;20 mM CaCl2; 50 mM HEPES, pH 7.5), following the protocol previously described Quick and Serrano (2007), Denver et al. (2009), and Muñoz et al. (2015). The embryos then developed until stage 40 or stage 46, in which the pallial evagination is in process or already completed, respectively. (2) To trace the fates of newly produced pallial cells BrdU was administered to stages 46 and 50, larval stages where the pallial evagination process is completed (**Table 1**), by immersion in 20 mM BrdU for 1 h at room temperature. The experimental animals were sacrificed at 1, 15, 30, and 60 days post-BrdU incorporation. Treatment regimens were adapted and optimized so that they resulted in similar natural mortality for experimental animals and controls. In each case the animals were additionally staged before processing. All the animals were anesthetized by immersion in a 0.3% solution of tricaine methanesulfonate (MS222, pH 7.4; Sigma–Aldrich, Steinheim, Germany) followed by immersion overnight at 4°C in MEMFA (0.1 M MOPS [4-morpholinopropanesulphonic acid], 2 mM ethylene glycol tetraacetic acid, 1 mM MgSO4, 3.7% formaldehyde). In the case of embryos and early larvae (until st 48) the brains were dissected out and were incubated for 30 min at 37°C with 2N HCl in PB to denature DNA strands and rinsed in PB. At these early stages the immunohistochemical procedure was carried out in toto: the brains were incubated overnight at room temperature with the monoclonal antibody mouse anti-BrdU (1:500; DSHB catalog reference: G3G4), followed by Alexa 488-conjugated goat anti-mouse (1:500; Molecular Probes; catalog reference A21042). Subsequently, the brains were blocked in gelatin and cut on a freezing microtome at 14–20 μm thick transverse sections. In the case of the late larvae (from stage 49) the brains were dissected out and cut on a freezing microtome at 18–30 μm thicknes and the sections were incubated for 30 min at 37°C with 2N HCl in PB to denature DNA strands and rinsed in PB just before the immunohistochemical procedure. Then the sections were incubated overnight at room temperature with mouse anti-BrdU (1:500; DSHB catalog reference: G3G4), followed by Alexa 488-conjugated goat anti-mouse (1:500; Molecular Probes; catalog reference A21042). The sections were coverslipped with fluorescence mounting medium, containing 1.5 μg/ml 4′,6-diamidino-2-phenylindole (DAPI) for DNA counterstaining (Santa Cruz; SC-24941 or Vectashield H-1500).

### Imaging

The analysis of the sections was made with a fluorescence microscope (Olympus BX51) equipped with appropriate filter combinations. Photomicrographs were obtained with a digital camera (Olympus DP72) adapted to the microscope. Additionally, sections were also analyzed with an Olympus FV 1200 or Leica sp-2 AOBS confocal microscope to assess the actual coexpression of two different markers in the same cell. Final adjustment of contrast and brightness of the photomicrographs was made with Adobe PhotoShop CS4 (Adobe Systems, San Jose, CA, USA). The compositions of the figures were mounted on plates using Canvas 11 (ACS Systems International, Santa Clara, CA, USA).

## Results

### Identification of the Pallial Boundaries during Development

During the embryonic and larval development, the pallium of *Xenopus* changes enormously, from the non-evaginated state to the full-evaginated hemispheres characteristic of the adult (**Table 1**). The identification of the pallial territories, mainly at early developmental stages, was achieved by the use of specific pallial versus subpallial markers. It should be noted that in *Xenopus* the embryonic development ends at stage 45 (4 dpf) when the animal starts feeding, and it is followed by a long larval period generally subdivided into three set of stages (see Bandín et al., 2013, 2015; Morona and González, 2013): (1) premetamorphosis (stages 46–52), which comprises the initial set of stages in which the larvae merely grow in size and early buds of the hind limbs start to be visible on the lateral side of the body; (2) prometamorphosis (stages 53–58), the period through which the hind limbs progressively develop; and (3) metamorphic climax (stages 59–65), period in which the tail of the tadpole is reabsorbed and leads to the tailless, four-legged froglet. In the brain of the recently metamorphosed juvenile (stages 65-66) all main anatomical characteristics as in the adult brain are already recognizable. We will refer to these larval periods in the description of the results.

**FIGURE 1:**
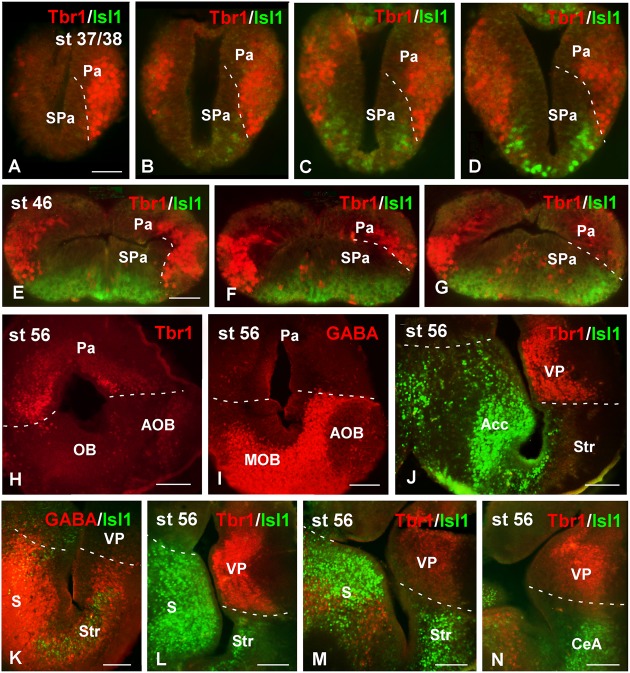
**Pallial boundaries during development**. Micrographs of transverse sections through the telencephalon of *Xenopus laevis* at embryonic **(A–D)**, premetamorphic **(E–G)** and prometamorphic **(H–N)** stages. In each panel the developmental stage and the color code for the used markers are indicated. In the developing telencephalon, the combined immunohistochemical detection of Tbr1, expressed in the pallium, and Isl1, a subpallial marker, clearly allowed the identification of the boundary between both regions throughout the rostrocaudal extent **(A–G,J,L–N)**. The localization of Tbr1 **(H)** at rostral level, in comparison with GABA **(I)**, highlights the olfactory bulb, where GABA was very abundant, in contrast to the pallium, where the Tbr1 expression was observed **(H,I)**. Simultaneous labeling for GABA and Isl1 discerns the SPa from the pallium **(K)**. Scale bars = 50 μm **(A–G)**, 100 μm **(H–N)**. See list for abbreviations.

To analyze the actual extent of the pallium at any developmental stage, Tbr1 expression in the pallium in combination with Isl1 expression in the SPa, served to highlight their boundaries (see Moreno et al., 2008a). GABA staining to discern the SPa from the pallium was also used (**Figure 1**). At early embryonic stages, the combination of Tbr1 and Isl1 labeling, analyzed from rostral to caudal telencephalic regions (**Figures 1A–D**), showed a distinct exclusive dorsal and ventral pattern, respectively, and the pallial territory appeared larger than the SPa. Starting by stage 46, the telencephalic vesicles became patent and both markers conserved the complementary dorso-ventral pattern of expression (**Figures 1E–G**). Actually, the Tbr1 expression indicated the ventral pallial limit, but it did not reach the Isl1 expression domain that avoided the most dorsal striatal portion (**Figures 1E–G**). At prometamorphic stages, the morphology of the hemispheres was recognized, where the main and AOBs were observed and discernible from the rest of the pallium by the lack of Tbr1 expression (**Figure 1H**), highlighting the bulbar boundaries, which could also be determined by the GABA staining (**Figure 1I**). More caudally, at these stages the lateral wall of the hemisphere develops a large striatal and ventropallial regions that could be individualized by the exclusive Tbr1/Isl1 staining combination (**Figure 1J**). The lack of Isl1 in the dorsal portion of the Str was observed by its combination with GABA, which labels densely packed cells in all the SPa and specially in this boundary region with the VP (**Figure 1K**). This pattern of staining is maintained through the most caudal levels of the telencephalon (**Figures 1L–N**).

### Pallial Ventricular Zone

**FIGURE 2:**
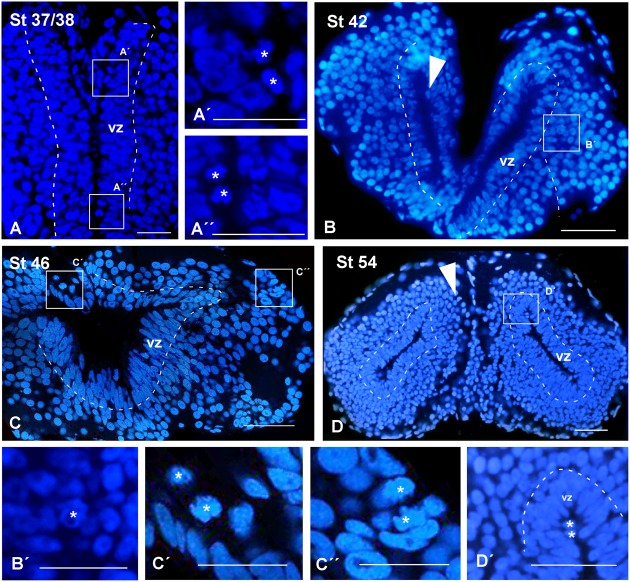
**Ventricular limits and mitotic cells**. Micrographs of transverse sections through the pallium of *Xenopus laevis* at embryonic **(A,A′,A′′,B,B′)**, premetamorphic **(C,C′,C′′)** and prometamorphic **(D,D′)** stages. The DAPI staining allowed the identification of the ventricular extent **(A–D)**, and, additionally, to examine the position of mitotic cells **(A′,A′′,B′,C′,C′′,D′). (A–C)** Are confocal images, and the higher magnifications **(A′,A′′,B′,C′,C′′,D′)** correspond to the framed areas, as indicated (white boxes in **A–D**). Throughout development, in ventricular layer the cells are densely packed, but the thickness of this layer was larger at early stages **(A,B)**, than later in larval stages **(C,D)**. At embryonic stages, ventricular mitotic cells were primarily visualized (**A′,A′′**, see arrowhead in **B**), but also abventricular mitotic cells were detected (see asterisk in **B′**). During the larval period, mitotic cells were also observed in the ventricular side (asterisk in **C′D′**) and abventricularly (asterisk in **C′′** and see arrowhead in **D**). Scale bars = 50 μm **(A–C,A′,A′′)**, 25 μm **(B′,C,C′′)**, 100 μm **(D,D′)**. See list for abbreviations.

At all stages of development, the sections used for the identification of other markers were systematically stained with DAPI for the localization of the chromatin content of the cells, allowing the analysis of the ventricular lining throughout development (see dashed white lines in **Figure 2**). At the early stages analyzed, the pallium showed a relatively thick vz (**Figure 2A**), which became relatively thinner during development (**Figures 2B,C**). In addition, the DAPI staining made it possible to localize mitotic cells within the pallium, since prophase, metaphase, anaphase, and telophase are easily identified. At the developmental stages analyzed, most mitotic cells in the pallium were located at the vz, both at embryonic (**Figures 2A′,A′′**, arrowheads in **Figure 2B**) and larval (**Figures 2C,C′,D,D′**) stages. However, mitotic cells were also identified away from the ventricle at all stages but were more abundant in late embryos (**Figure 2C**′′, arrowheads in **Figure 2D**).

### Mitotic Rate

**FIGURE 3:**
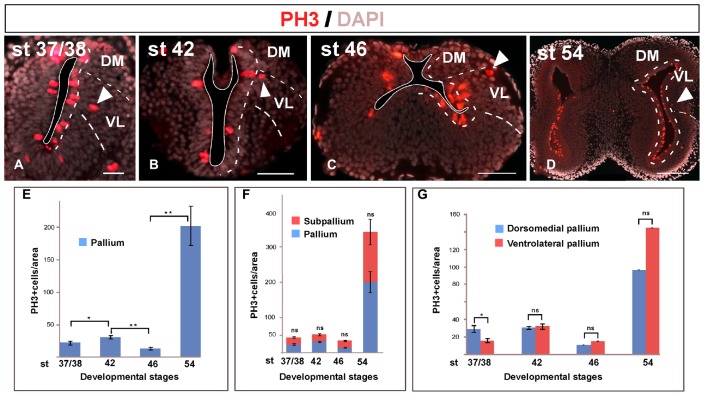
**Mitotic rate**. Micrographs of transverse sections through the pallium of *Xenopus laevis* at embryonic **(A,B)**, premetamorphic **(C)** and prometamorphic **(D)** stages showing the distribution of PH3, marker of proliferating and mitotic cells, in combination to DAPI. During development, mitotic cells were observed in the ventricular cell layer, but also abventricularly (arrowheads in **A–D**). The mitotic index significantly increased at the end of the embryonic period, but it significantly decreased at early larval stages, to drastically increase later at stage 54, when a neurogenetic peak is observed **(E)**. This pattern is observed not only in the pallium, but also when pallial and subpallial regions separately analyzed, where significant differences in their mitotic index were not observed at any stage **(F)**. Considering separately during development the DM and the VL, major differences in their mitotic index were not observed, with the exception of the stage 37/38 when the dorsomedial proliferation is slightly higher, but it did not continue later in development **(G)**. Scale bars = 50 μm **(A–C)**, 200 μm **(D)**. See list for abbreviations. ^∗∗^*P* < 0.001, ^∗^*P* < 0.05.

The phosphorylated form of histone H3 (PH3) is present in the cell division along the late G2 phase and in the M phase (Hendzel et al., 1997). The presence of PH3 is commonly used for the identification of mitotic cells during proliferation (von Bohlen und Halbach, 2011). The PH3 analysis allowed confirming that mitotic cells were mainly situated in the vz but not exclusively (arrowheads in **Figures 3A–D**). In addition, the PH3 staining allowed us to analyze the proliferative rate of the telencephalon during development (**Figures 3E–G**).

The analysis of the pallial mitotic index from embryonic to late larval stages showed that it drastically and significantly varied along development (**Figure 3E**). At the end of the embryonic period, the comparison between stages 37/38 and 42 showed that the mitotic index significantly increased. However, it significantly decreased when the long larval period starts, at stage 46 (**Figure 3E**). The animals are kept at this low mitotic rate during a variable period of time, and in many cases it was observed that many tadpoles stopped their development retaining external features of mid larval stages, and eventually dying. Later in larval development, the mitotic index drastically increased at stage 54, when a neurogenetic peak is reached, in sharp contrast to the quiescence observed earlier at stage 46 (**Figure 3E**). When we analyzed if these differences were due to a specific telencephalic region, we observed that when pallial and subpallial regions were separately analyzed the mitotic rates were comparable and significant differences were not observed between the pallium and the SPa (**Figure 3F**). Similarly, when the mitotic rate was analyzed at different stages in the DM versus the VL, significant differences were not observed (**Figure 3G**), with the exception of the stage 37/38 when the dorsomedial proliferation was slightly higher, but it was not maintained in later development. Thus, the mitotic index detected later in development was comparable between the dorsomedial and the ventrolateral regions at both neurogenic periods observed, i.e., at the quiescence phase and at the posterior neurogenetic peak observed at stage 54 (**Figure 3G**).

### BrdU Assays

**FIGURE 4:**
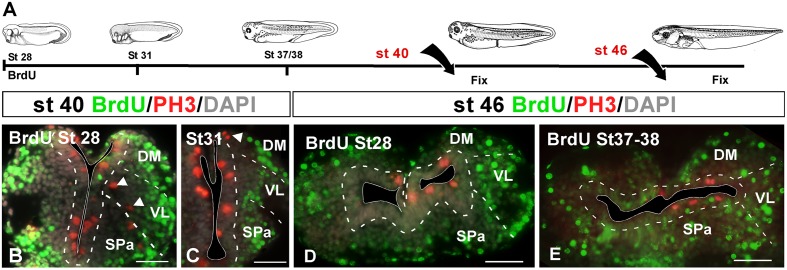
**Proliferation analysis with BrdU. (A)** Schematic drawing illustrating the experimental approach used, in which BrdU was administrated at the embryonic stages specified and the animals were sacrificed at the larval stages indicated. **(B–E)** Micrographs of transverse sections through the pallium of *Xenopus laevis* at embryonic **(B,C)** and premetamorphic larvae **(D,E)** stages showing the distribution of BrdU labeling in combination to PH3 and DAPI; the color code and the stages analyzed are indicated. The developmental stage of BrdU administration is indicated in each micrograph. After the BrdU administration at early embryonic stages and subsequent processing at late embryonic stages **(B,C)** and early larvae stages **(D)**, the BrdU+ cells were situated in the mantle zone equally distributed and double PH3/BrdU labeled cells were not detected **(B,C)**, but PH3 mitotic cells in ventricular and abventricular positions were observed (see arrowheads in **B,C**). After the BrdU administration at late embryonic stages and subsequent processing at early larvae stages **(E)** there were not PH3/BrdU+ double labeled cells, and the BrdU+ cells were situated in the mantle zone, but additionally in the ventricular layer. Scale bars = 50 μm **(B–E)**. See list for abbreviations.

In a first experimental approach, BrdU was administrated at embryonic stages 28, 31, and 37/38, in which the telencephalic vesicles were not yet evaginated. They were then processed at stage 40, when telencephalic evagination is in course, or at stage 46 when the evaginated hemispheres are formed (**Figure 4A**). In all cases, sections were further processed for PH3 in order to identify mitotic cells at the time of fixation. When the BrdU was administrated at stage 28 and 31, later at stage 40 the BrdU+ cells were mainly situated laterally (superficially) in the mantle zone, homogeneously distributed, and there were not double PH3/BrdU labeled cells (**Figures 4B,C**). The PH3-ir cells were mainly situated in the vz and occasionally labeled cells were also detected away from the ventricle (arrowheads in **Figures 4B,C**). Similarly, when BrdU was administrated at stage 28 and the animals were maintained until larval stage 46, the BrdU+ cells were found uniformly distributed in the mantle zone, always avoiding the vz, and double labeled cells for PH3 were never found (**Figure 4D**). Animals in which BrdU was administrated at stage 31 and processed at stage 46 showed a pattern similar to the case described above (data not shown). Finally, animals fixed at stage 46 after BrdU administration at stage 37/38, near the start of the telencephalic evagination, the BrdU+ cells were never PH3-ir and were randomly and laterally distributed. In contrast to earlier stages analyzed, in this case more BrdU labeled cells were observed in the vz, however, they did not coexpress PH3 (**Figure 4E**).

**FIGURE 5:**
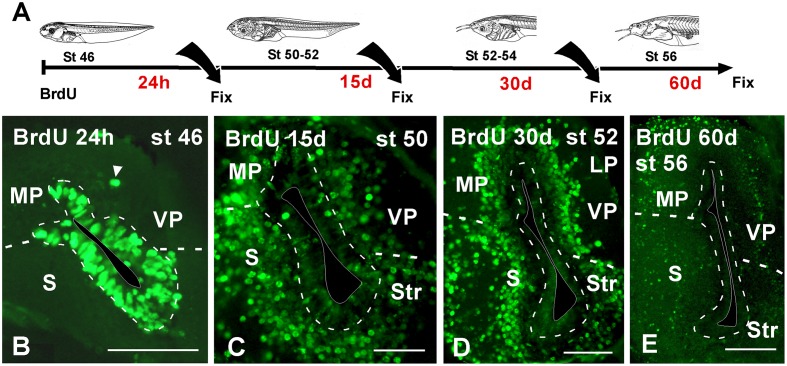
**Neuronal birth during pallial development. (A)** Schematic drawing illustrating the experimental approach used, in which BrdU was administrated at the stages specified and the animals were sacrificed at the larval stages indicated. **(B–E)** Micrographs of transverse sections through the pallium of *Xenopus laevis* at premetamorphic **(B)**, prometamorphic **(C,D)** and metamorphic **(E)** stages. Twenty four hour after the BrdU administration almost all the positive cells were concentrated in the ventricular zone **(B)** and additionally abventricular BrdU+ cells were found (arrowhead in **B**). 15 days after the BrdU, labeled cells were found away from the ventricle in adjacent layers **(C)**. Similarly, 30 days **(D)** and 60 days **(E)** after the administration, the BrdU+ cells migrated to more superficial positions. Scale bars = 100 μm **(B–E)**. See list for abbreviations.

To better understand patterns of cell migration in the developing pallium, in a second set of experiments BrdU was administrated at stages 46 and 50, when the telencephalic vesicles are evaginated, and then animals were allowed to develop for 24 h, 15, 30, and 60 days before processing (**Figure 5A**). For simplification, in the schematic representation (**Figure 5**) the data refer to the results obtained in the stage 46, but in both cases the results were totally comparable. When the animals were processed 24 h after the BrdU administration, many of the ventricular cells were BrdU+ and were homogeneously distributed in the pallium (**Figure 5B**). In addition, BrdU+ cells were also observed separated from the vz (arrowhead in **Figure 5B**). The animals that were processed 15 days later, when the larvae reached premetamorphic stages, the BrdU labeled cells had left the ventricular lining and occupied adjacent cell layers, randomly distributed throughout the rostrocaudal and mediolateral pallial extent (**Figure 5C**). Later in development, the location of BrdU+ cells after 30 (**Figure 3D**) and 60 days (**Figure 3E**) was coincident, and they occupied distant superficial positions in all pallial subdivisions, rostrocaudally and mediolaterally, consequently following the outside-in order detected earlier.

### Identification of Progenitor Cells

**FIGURE 6:**
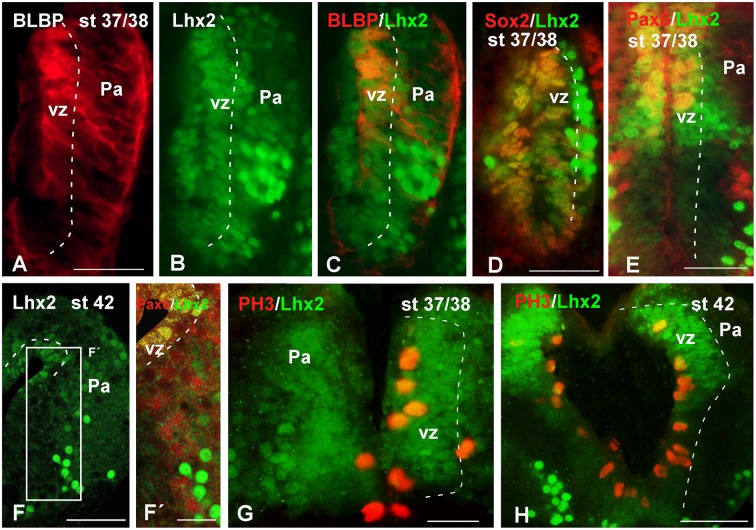
**Analysis of progenitor cells at embryonic stages (BLBP, Lhx2, Sox2, Pax6, PH3)**. Micrographs of transverse sections through the pallium of *Xenopus laevis* at embryonic stages showing the codistribution of the progenitor markers BLBP **(A,C)**, Lhx2 **(B,C,E–G)**, Sox2 **(D)**, and Pax6 **(E,F′)**; and the combination of Lhx2 with the mitotic marker PH3 **(G,H)**. In each panel the developmental stage and the color code for the used markers are indicated. **(F,F′)** Are confocal images, and the higher magnification shown in **(F’)** corresponds to the framed area indicated in **(F)**. The BLBP **(A)** and the Lhx2 **(B)** labeling detected at stage 37/38 showed double labeled cells exclusively in the ventricle **(C)**. The combination of Lhx2 and Sox2 also showed double labeled cells in the ventricle, but the cells detected for both markers in the mantle were never double labeled **(D)**. In the case of Pax6, Lhx2/Pax6 double labeled cells were observed in the ventricular zone **(E,F′)**, but never away from this region, where both cell populations were intermingled **(F′)**. The labeling of Lhx2 in combination with PH3 showed that only the ventricular cells expressing Lhx2 were mitotic cells **(G,H)**. Scale bars = 50 μm. See list for abbreviations.

**FIGURE 7:**
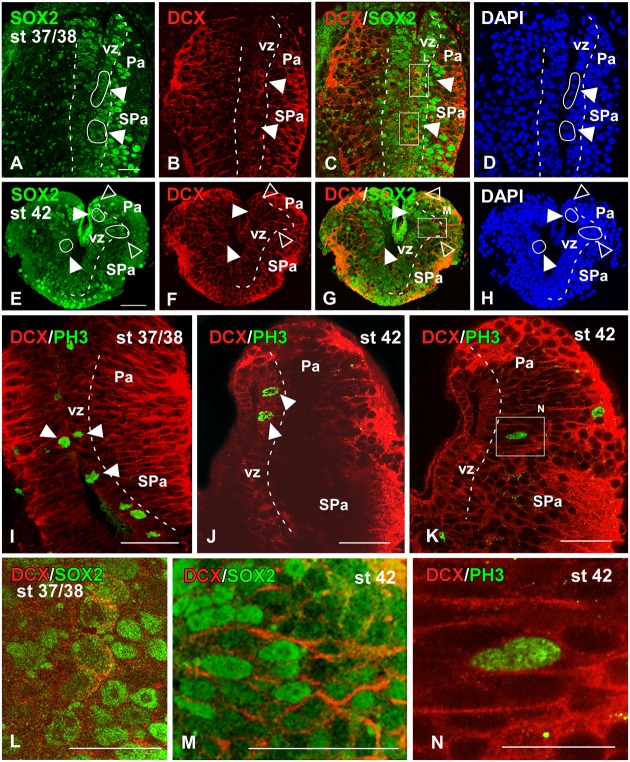
**Analysis of progenitor cells at embryonic stages (Sox2, DCX, PH3)**. Micrographs of transverse sections through the pallium of *Xenopus laevis* at embryonic stages 37/38 **(A–D,I,L)** and 42 **(E–H,J,K,M,N)** showing the codistribution of the progenitor marker Sox2 **(A,E)** and the neuroblast marker DCX **(B,F)** in combination to DAPI **(D,H)**, and the combination of DCX with the mitotic marker PH3 **(I–K)**. In each panel the developmental stage and the color code for the used markers are indicated. All micrographs are confocal images, and the higher magnifications **(L,M,N)** correspond to the framed areas, as indicated (white boxes in **C,G,K**). At the ventricular zone there were observed double labeled cells for Sox2 and DCX (see arrowheads in **A–C, E–G**), and those cells were mitotic cells (see white circles and filled arrowheads in **D,H**). Additional double labeled cells were observed away from the ventricle (see empty arrowheads in **E–H**). The combination of DCX and PH3 showed double DCX/PH3 labeled cells in the ventricle and away from it (see arrowheads in **I,J**). Scale bars = 25 μm **(A–H,L,M)**, 50 μm **(I–K)**. See list for abbreviations.

**FIGURE 8:**
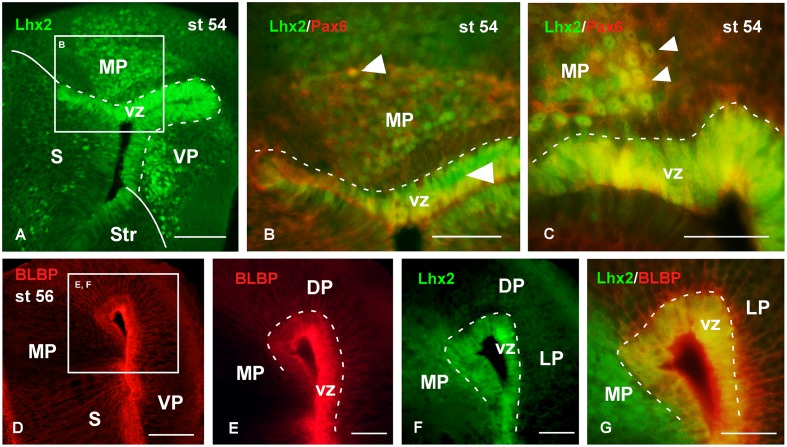
**Analysis of progenitor cells at larval stages (Lhx2, Pax6, BLBP)**. Micrographs of transverse sections through the pallium of *Xenopus laevis* at the larval stage 54 showing the distribution of the progenitor markers Lhx2 **(A–C,F,G)**, Pax6 **(B,C)** and BLBP **(D–G)**. In each panel the color code for the used markers is indicated. The higher magnifications **(B,E,F)** are from the panels indicated in each case (white boxes in **A,D**). At this stage in the ventricular zone Lhx2 is expressed by virtually all cells **(A,F)**, and those cells co-expressed Pax6 **(B,C)** and BLBP **(G)**. Lhx2 expressing cells away from the ventricle, like in the medial pallium, were also labeled for Pax6 **(B,C)**, but never expressed BLBP **(G)**. Scale bars = 100 μm **(A,D)**, 50 μm **(B,C,E–G)**. See list for abbreviations.

**FIGURE 9:**
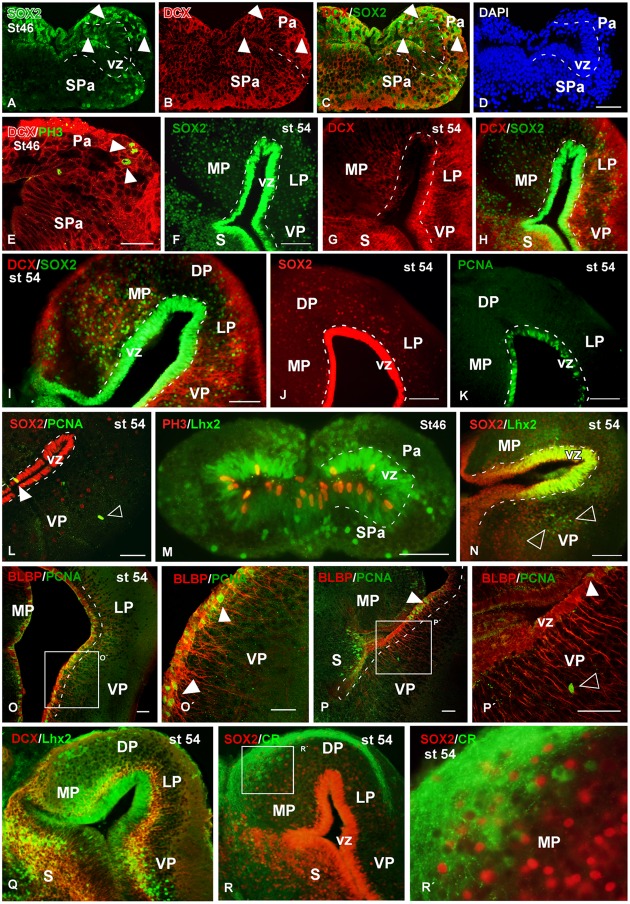
**Analysis of progenitor cells at larval stages (Sox2, DCX, BLBP, PCNA, PH3)**. Micrographs of transverse sections through the pallium of *Xenopus laevis* at larval stages 46 **(A–E,M)** and 54 **(F–L,N–R)** showing the distribution of the progenitors markers Sox2 **(A,C,F,H,I,J,L,N)**, the neuroblasts marker DCX **(B,C,E,G–I,Q)** and the progenitors markers Lhx2 **(M,N,Q)** and BLBP **(O,P)**, and their combinations with the mitotic marker PCNA **(L,O,P)** and PH3 **(M)**. In addition, the codistribution of DCX and calretinin is shown **(Q)**. In each panel the developmental stage and the color code for the used markers are indicated. **(A–E,O,P)** Are confocal images, and the higher magnifications **(O′,P′,R′)** correspond to the framed areas, as indicated (white boxes in **O,P,R**). At stage 46 Sox2/DCX double labeled cells were observed in the ventricular zone and away from it (arrowheads in **A–C**), and DCX labeled mitotic cells away from the ventricle were observed (arrowheads in **E**). At stage 54 Sox2 is expressed in the ventricular and mantle zones, and cells that coexpressed DCX were observed **(F–I)**. Close to the ventricle those Sox2 cells were mitotic cells **(J,K)**, but the Sox2 expressing cells detected away from the ventricle were not mitotic (empty arrowhead in **L**). The Lhx2 expressing cells detected at larval stages were mitotic in the ventricle **(M)** and coexpressed Sox2 **(N)**, but in the mantle zone they were not coexpressing PH3 **(M)** and were intermingled with the Lhx2 cell population (empty arowheads in **N**). The combination of BLBP and PCNA showed that the mitotic cells in the ventricular zone coexpressed BLBP (arrowheads in **O′,P,P′**), but not those separated from the ventricle (empty arrowhead in **P′**). The combination of Lhx2 and DCX showed that the Lhx2 cells detected close to the adjacent vz were double labeled **(Q)**. The combination of Sox2 to calretinin, a marker of a population of pallial interneurons, showed that there were not double labeled cells **(R,R′)**. Scale bars = 50 μm **(A–E,L,M,O–P′)**, 200 μm **(I)**, 100 μm **(F–K,N)**. See list for abbreviations.

**FIGURE 10:**
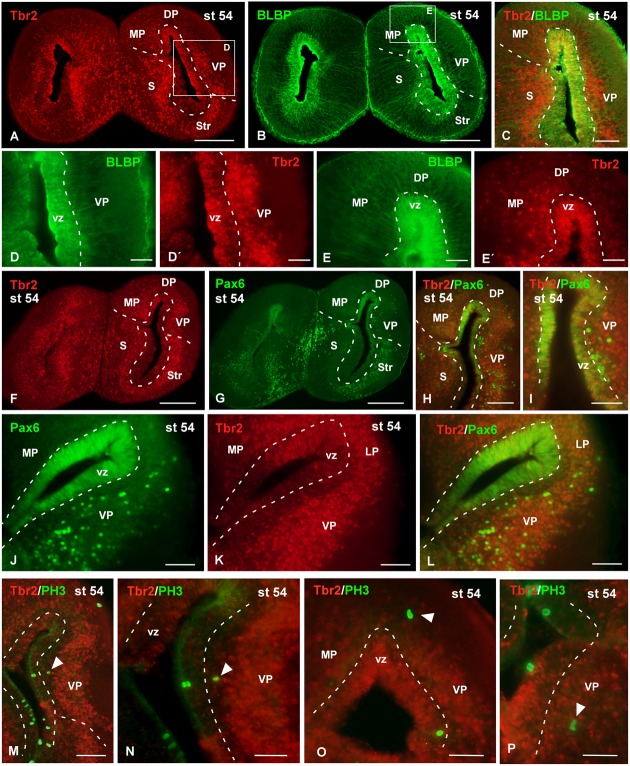
**Analysis of progenitor cells at larval stages (Tbr2, BLBP, Pax6, PH3)**. Micrographs of transverse sections through the pallium of *Xenopus laevis* at the larval stage 54 showing the distribution of the markers Tbr2 **(A,C,D′,E′,F,K)**, BLBP **(B,C,D,E)** and Pax6 **(G–J,L)**, and the combination of Tbr2 with the mitotic marker PH3 **(M–P)**. In each panel the color code for the used markers is indicated. The higher magnifications **(D,E)** correspond to the framed areas, as indicated (white boxes in **A,B**). Tbr2 expressing cells were detected close to the ventricle and away from it **(A,D′,E′,F,K)** but only those cells in the ventricular zone expressed BLBP **(D,D′,E,E′)** or Pax6 **(F–L)**. The combination of Tbr2 and PH3 showed that there are not Tbr2 mitotic cells away from the ventricle **(M–P)**. Scale bars = 200 μm **(A,B,F,G)**, 100 μm **(C,H,J–M)**, 50 μm **(D,E,I,N,O,P)**. See list for abbreviations.

With the aim of characterizing the progenitor cells in the *Xenopus* pallium from embryonic to late larval stages (**Figures 6**–**10**), we used a BLBP antibody as a marker of RGc-like progenitor cells (Brunne et al., 2010), in combination with Lhx2 used as marker of pallial precursor cells. At stages 37/38, BLBP-ir cells were found in the vz and showed cell processes into the mantle (**Figure 6A**). In the case of Lhx2, the labeled cells were observed in the vz and, additionally, separated from the ventricle. Of note, those cells in the mantle zone showed a higher intensity in the labeling than those located in the vz (**Figure 6B**). When we combined both markers, the double Lhx2/BLBP-ir cells were located exclusively in the vz, and the Lhx2-ir cells found in the mantle never expressed BLBP (**Figure 6C**). Furthermore, precursor cells were also identified by the expression of Sox2, expressed in proliferating precursor cells and in glial-like cells that are stem cells. At these stages, the Sox2-ir proliferative precursor cells were restricted to the vz and coexpressed Lhx2, but cells separated from the vz were not found to coexpress both markers (**Figure 6D**). In the case of Pax6, a marker of pallial precursors, the vz cells showed low expression but when the labeled cells were postmitotic the intensity increased, and only the vz Pax6-ir cells coexpressed Lhx2, both at stages 37/38 (**Figure 6E**) and later at stage 42 (**Figures 6F,F**′). Finally, at stages 37/38 and 42 the PH3 labeling showed Lhx2-ir mitotic cells in the vz (**Figures 6G,H**).

During the early embryonic period, the combined immunodetection of Sox2 and DCX that characterizes neuroblast cells and young neurons (von Bohlen und Halbach, 2011) allowed us to observe that virtually all cells in the vz were Sox2-ir and only scarce cells were labeled separated from the ventricle (**Figure 7A**). In turn, DCX-ir cells were primarily located in peripheral (superficial) location although sparsely labeled cells were also observed in the vz (**Figure 7B**), which were also labeled for Sox2 (**Figure 7C**) and were identified as mitotic cells by the DAPI staining (arrowhead in **Figures 7A,D,L**). Later in development, by embryonic stage 42, the Sox2-ir cells were still numerous in the vz (**Figure 7E**) and, in addition to the Sox2/DCX-ir cells in the vz (filled arrowheads in **Figure 7G**) some double labeled cells were also noted outside the vz (empty arrowheads in **Figures 7E–H**). However, the Sox2/DCX-ir cells that were separated from the ventricle only occasionally showed mitotic features. These results were corroborated by the combination of DCX with PH3, which identifies proliferative cells (**Figures 7I–K**). In this case, double DCX/PH3-ir cells were found in the vz at embryonic stages (arrowheads in **Figures 7I,J**) and outside the vz in more superficial regions (**Figures 7K,N**). Of note, PH3-ir cells outside the vz that did not express DCX were also constantly observed among DCX-ir cells (**Figure 7K**).

The analysis of the localization of the progenitor cells was also conducted throughout the larval period (**Figures 8**, **9**). Thus, at stage 54 it was found that virtually all cells in the vz expressed Lhx2 (**Figure 8A**), and mainly those actually lining the ventricle coexpressed Pax6 (**Figures 8B,C**). Interestingly, Lhx2 expressing cells were also relatively abundant primarily in the mantle zone of the medial and ventral pallial regions (**Figure 8A**). Pax6 expressing cells were also observed in the mantle zone but they did not reach as far superficially as the Lhx2-ir cells. Only in the medial pallium some of the cells separated from the vz were observed to coexpress Lhx2 and Pax6 (**Figure 8B**). Regarding the pattern of BLBP staining at similar larval stages, it was found that most vz cells expressed this marker, which allowed the observation of fine, radially oriented processes (**Figures 8D,E**). The BLBP staining in combination with Lhx2, at stage 54, demonstrated that most cells in the vz were double labeled in the medial pallium, whereas the Lhx2-ir cells separated from the ventricle did not contain BLBP (**Figure 8G**).

At larval stages, the Sox2 expressing progenitor cells were also identified in the vz and also abundantly dispersed into the mantle (**Figures 9A,F,I,J,L,N**). Colocalization of Sox2 and DCX was observed in cells of the vz in early and late larval stages (**Figures 9C,H,I**). In addition, Sox2/DCX double labeled cells were also detected far from the ventricle. Furthermore, it was corroborated that many Sox2-ir vz cells were mitotic cells (**Figure 9E,J,K,L**). Also at these stages, Lhx2 and Sox2 staining was obtained in the vz, where mitotic cells expressing these markers were also labeled for PH3 (**Figure 9M**). In contrast, the Lhx2 expressing cells located away from the vz, which were very abundant at these larval stages, were never found to express Sox2 (empty arrowheads in **Figure 9N**). Sox2 expression analyzed in combination with calretinin (marker of a mature interneuron population in the *Xenopus* pallium; Morona and González, 2008, 2013) demonstrated lack of colocalization (**Figures 9R,R**′). Finally the analysis at larval stages of BLBP expression in RGc demonstrated the ventricular position of these cells (**Figures 9O,P**). In addition, when PCNA and BLBP double labeling was conducted, it was found that all the proliferating cells detected in the vz expressed BLBP (arrowheads in **Figures 9O,P**′), in contrast to the abventricular PCNA-ir cells that did not express BLBP (empty arrowhead in **Figure 9P**′). The combined observation of Lhx2 and DCX at the larval stage 54 highlighted double labeled cells in the subventricular zone (svz), close to the adjacent vz (**Figure 9Q**).

Attending to the analysis of the Tbr2-ir cells at larval stages it was observed that the cells that expressed this marker were widely distributed in the pallium (**Figure 10**). The combined staining for BLBP showed that the Tbr2-ir cells were located in the vz and subventricular region (**Figures 10A–E**′). In general, Tbr2 cells were abundant but scattered distributed, with the exception of the VP where the labeled cells constituted masses that formed nuclei-like aggregations (**Figures 10D′,K**). By means of Pax6 staining it was observed that no double Tbr2/Pax6 labeled cells separated from the vz (**Figures 10F–I**). Furthermore, the analysis of the PH3 labeling demonstrated that Tbr2-ir cells situated outside the vz were not mitotic cells, at any rostrocaudal level of the telencephalon (**Figures 10M–P**).

## Discussion

The present study aims to contribute to the understanding of the different aspects of the formation of the telencephalic pallium in a comparative perspective. One of the focuses in the study of the evolutionary differences of this region has been its neurogenesis, that is, the mechanisms of development by which neurons are generated from neural progenitor cells. This process includes many aspects such as proliferative capacity, number of progenitors and their cell division types, proliferation rates, cell cycle length or the lineage relationships of different types of progenitors, which vary in the different vertebrate models (Kornack and Rakic, 1998; Nomura et al., 2013a, 2016; Florio and Huttner, 2014; Martínez-Cerdeño et al., 2016; Montiel et al., 2016).

The analysis of the pallium throughout development in the anuran amphibian *Xenopus laevis* (anamniote tetrapod) serves to shed light on the anamniote-amniote transition, since particular attention has been given only to reptiles and birds in recent years (Nomura et al., 2013a,b, 2015, 2016; Suzuki and Hirata, 2014; Martínez-Cerdeño et al., 2016; Montiel et al., 2016). Although general patterns of cell proliferation in the anuran brain have been studied (Wullimann et al., 2005; Chapman et al., 2006; Raucci et al., 2006; Simmons et al., 2006; Coen et al., 2007; Denver et al., 2009; Tao et al., 2015; Thuret et al., 2015), no detailed analysis of the pallium has been performed. We have selected some markers of the main proliferative events described in other vertebrates (PH3, PCNA and BrdU assays), which had previously been successfully used in *Xenopus* (D’Amico et al., 2011, 2013; Auger et al., 2012; McKeown et al., 2013) and allowed us to conduct birth-dating and mitotic rate analysis.

Similar approaches to those used in mammals were also used in *Xenopus* for the analysis of pallial progenitor cells. It should be noted, prior to discussion, that the terminology used by different authors to define different progenitor cells varies considerably. Cortical development has mostly been studied in rodent animal model systems, that is, mouse and rat. In general, the germinal zone harboring progenitors that have apical junctions facing the lumen of the lateral ventricles and that undergo mitosis at this apical surface are referred to as RGc or apical progenitors, which can be visualized by the expression of vimentin and/or BLBP. BLP most likely represents a good marker for RGc-like progenitors because it is not expressed in cells that express mature astrocyte markers or in cells expressing markers of the neuronal lineage (Brunne et al., 2010). These cells can also be identified by the Pax6 expression, because this transcription factor is necessary to maintain the radial glial identity (Noctor et al., 2004; Martínez-Cerdeño et al., 2006, 2012) and Lhx2 (Chou and O’Leary, 2013; Bery et al., 2016). In turn, the surface toward the pia is termed outer or basal, and those progenitor cells that characteristically undergo mitosis in a secondary germinal zone situated basal to the vz and named the svz, are termed IPs or basal progenitors. These cells in rodents down-regulate Pax6 and express Tbr2 (Martínez-Cerdeño et al., 2012, 2016). Tbr2 expression is gradually reduced as the cells progressively commit to the neuronal lineage and leave the mitotic cycle (Hodge et al., 2008), whereas other genes such us Tbr1 are up-regulated. In contrast, IPs in the developing primate neocortex may sustain Pax6 expression, in line with their increased proliferative capacity (Betizeau et al., 2013). Sox2 is intensely expressed in RGc, whereas it is only found at low levels in IPs (Hutton and Pevny, 2011). Finally, DCX expression characterizes neuroblasts during migration and young neurons (Francis et al., 1999; Gleeson et al., 1999), and the expression of DCX is associated with a central phase of neurogenesis. This phase comprises from the progenitor stage to the stage when functional connections are made by dendrites and axons of the newly generated cells, which start expressing adult markers like the calcium binding protein calretinin (Knoth et al., 2010). Therefore, the localization of DCX expression is specifically related to newly generated neurons, since virtually all cells that express DCX also express early neuronal antigens but lack specific markers for glial, undifferentiated, or apoptotic cells (Rao and Shetty, 2004).

### Pallial Neurogenic Periods: Rates of Proliferation

**FIGURE 11:**
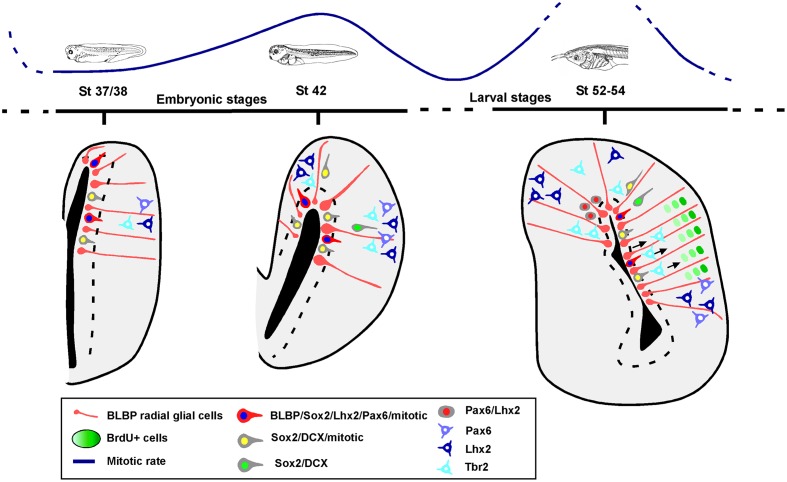
**Summary diagram of the progenitor cells in the pallium of *Xenopus laevis***. Schematic representations showing that there are two phases of progenitor cells divisions, at mid-embryonic period and at mid-larval development. The pallial developmental order follows an outside-in pattern, in which BLBP, Pax6, Lhx2 and Sox2 are expressed in the ventricular proliferative zone and later Lhx2 and Pax6 are expressed in postmitotic cells away from the ventricle. Sox2 mitotic cells are present in ventricular and abventricular zones, and some of those cells express DCX. In the case of Tbr2, it is not expressed by mitotic abventricular cells.

The analysis of the mitotic rate has been performed by identifying the cells expressing PH3 and the results lead to the recognition of distinct neurogenic periods in the pallium of *Xenopus*. Throughout the stages analyzed, the proliferative rates vary and there is a significant quiescence period around the initial larval period, at stage 46, which changes dramatically in later prometamorphic stages, at stage 54, when a high neurogenetic peak is detected (results summarized in **Figure 11**).

Comparatively, the current results in the telencephalon of *Xenopus* with previous studies in other brain regions, such as the optic tectum, the number of dividing precursor cells along the tectal ventricular layer decreased through development from stage 46 to stage 49 (Tao et al., 2015), which is largely similar to the observations in the pallium, specially in the pallium versus SPa. These results concur with similar data reported in the spinal cord (Thuret et al., 2015). Interestingly, in a study of the effect of thyroid hormone T3 on cell proliferation, it was observed that the number of proliferating cells during premetamorphosis and early prometamorphosis was similar in different brain regions (stages 52–55), with an increase in the number of proliferative cells later in development (Denver et al., 2009). In addition, it was also described that at stages 50–54 the *Xenopus* larvae show a strong regenerative capacity, which is no longer present later, probably related to the number of dividing precursor cells (Muñoz et al., 2015).

The length of the neurogenic period has been the focus of many evolutionary analyses over the last few years (reviewed in Florio and Huttner, 2014), since this parameter can determine the production of neurons and then contribute to understanding the differences between species. For example, the onset of neurogenesis has been reported to be delayed in primates (Kornack and Rakic, 1998), where furthermore neurogenesis itself extends for a much longer period of time, in both cases compared to rodents (Caviness and Takahashi, 1995; Rakic and Caviness, 1995). Therefore, it allows a progenitor expansion and, thus, a pallial enlargement in the lateral dimension, key in evolutionary terms. However, longer neurogenic periods would result only in an increase of neuron formation, if it is not accompined by an increase in the duration of the cell cycle. In this regard, it was recently observed in *Xenopus* that the cell cycle is gradually lengthened to reach a maximum length in stage 50, and during secondary neurogenesis (at step 54) a shortening of the cell cycle occurs (Thuret et al., 2015), thus coinciding with the quiescence versus neurogenic periods also observed in the pallium (current results). This is an interesting result because, for example, the frequency of cell divisions in the cortex of geckos is significantly lower than in other amniotes (Nomura et al., 2013a). Therefore the cell cycle length can influence the RGc pool and its variability across vertebrates might constitute one of the important evolutionary differences. In the particular case of *Xenopus*, the quiescence period observed throughout the larval development (Thuret et al., 2015; present results) coincides with the period when the “external” development of the larvae is highly variable and occasionally slow. Thus, during the larval stages there is high variability in the developmental timing, between animals of the same age (personal observations). There are specimens transiting this phase rapidly without stopping times, but there are many others that remain at an unpredictable stage for long periods of time without apparent variations in their size or external morphological features and, significantly, under similar ambient conditions of light, temperature, etc. If this is related to the quiescence period detected in the brain neurogenesis and thus to the length of the cell cycle, it should still be analyzed.

In mammals, during cortical neurogenesis it has been described that thalamic afferents influence early stages of corticogenesis (Molnar and Blakemore, 1995) by secreting diffusible factors that promote the proliferation of cortical precursors over a restricted developmental window, shortening the cell-cycle duration and increasing the number of proliferative divisions (Dehay et al., 2001). In fact, it was shown that in the developing ferret following binocular enucleation, in which the number of thalamocortical axons that reach the visual cortex decrease severely, the resulting visual cortex is reduced by a reduction of progenitor proliferation (Reillo et al., 2010; Reillo and Borrell, 2011). From an evolutionary point of view, the lack of the massive thalamic cortical input described in other vertebrates contrasts with the situation found in mammals. In the case of *Xenopus*, thalamic inputs to the pallium are especially restricted (ten Donkelaar, 1998; Moreno and González, 2004; Moreno et al., 2012). However, there are differences between pallial regions and, although they are scarce, there are thalamic projections to the medial and VP; regions considered counterparts in anurans of the hippocampus and pallial amygdala, respectively (Northcutt and Ronan, 1992; Moreno and González, 2004, 2007). It is interesting to note that these two regions were the pallial zones showing more prearranged svz (present results). The size of the svz and its complexity has been related to the acquisition of gyrencephalic cortices in mammals (Kriegstein et al., 2006; Reillo et al., 2010; Namba and Huttner, 2016). Moreover, in evolutionary terms it seems to be related to the formation of distinct layers in the pallium; at least one could postulate that in the species in which the svz is not subdivided and the existence of Tbr2+ IPs is doubtful (Nomura et al., 2016) no pallial layers are formed (see discussion below, Martínez-Cerdeño et al., 2016; Nomura et al., 2016; present results).

Finally, while adult mammals have limited proliferation and neurogenetic capabilities, *Xenopus* possesses high rates of proliferation and generation of new cells after the larval development is completed. Actually, several previous studies, using different markers of proliferation (PCNA, 3H thymidine, or BrdU) for the analysis of different brain regions, have demonstrated adult neurogenesis in anuran amphibians, primarily in species other than *Xenopus laevis* (Bernocchi et al., 1990; Chetverukhin and Polenov, 1993; Margotta et al., 2005; Wullimann et al., 2005; Raucci et al., 2006; Almli and Wilczynski, 2007; D’Amico et al., 2011).

### Radial Migration: Inside-Out versus Outside-In Order

One of the most obvious difference between mammalian and non-mammalian pallia concerns the formation of laminae. Thus, in mammals most parts the pallium are laminated and, in particular, the neocortex is characteristically a six-layered structure throughout its extension, although some variations exist between distinct areas and species. In contrast, the non-mammalian pallium is mainly organized in a non-laminar fashion, and the evolutionary origin of the neocortical layers and the cytoarchitectural boundaries with the SPa have been the focus of numerous studies. Thus, it has been largely debated in the last decades the question of how the six-layer neocortex arose from a “three-layered” situation of the DP in archosauria (a group of diapsid amniotes whose living representatives consist of birds and crocodiles), and even the earlier “non-layered” condition of the pallium in anamniotes. In this evolutionary context, amphibians are the only group of tetrapod anamniotes, which possess the four pallial subdivisions described in all amniotes (reviewed in Moreno and González, 2006, 2007). But the pallium on amphibians shows no signs of lamination (current results), in contrast to sauropsids, where at least three layers are present in some regions (reviewed in Medina and Abellán, 2009). This implies that the morphogenesis of the distinct pallial subdivisions is substantially different, although there is a common initial patterning and formation of major subdivisions, shared by the pallium of tetrapod vertebrates.

With respect to layer formation, one of the traditionally followed hypotheses was that the total absence of this layered structure in the pallia of non-mammalian vertebrates might suggest that that was the condition of the non-mammalian ancestors, and the layered neocortex represents an acquisition during early mammalian evolution (Northcutt and Kaas, 1995). Actually, the notion of this new addition is implied by the coined term “neocortex.” However, recent studies on the expression patterns of neocortical genes in the brains of non-mammalian vertebrates have challenged this interpretation that the neocortex is an added structure only in the evolution of mammals (reviewed in Medina and Abellán, 2009; Puelles, 2011; Puelles et al., 2016). It has been shown that the distinctive pattern of combinatorial expression for many transcription factors during mammalian development establishes the neocortical fate of the dorsal telencephalon. Strikingly, numerous studies are currently demonstrating that during the development of pallium of non-mammalian vertebrates, the same combination of transcription factors is expressed (Smith-Fernández et al., 1998; Puelles et al., 2000, 2016; Brox et al., 2004; Medina and Abellán, 2009; Suzuki and Hirata, 2011, 2013, 2014). This evidence strongly suggests that the non-mammalian pallium contains a homologous field of the mammalian neocortex. Actually, it was demonstrated that these homologous neocortical regions are used by mammals and birds for comparable cognitive and behavioral functions (Northcutt and Kaas, 1995; Medina and Reiner, 2000; Jarvis et al., 2005; Kaas, 2008), in spite of the evident differences in terms of histology. Future research should clarify how these animals reach the distinct species-specific pallial cytoarchitecture using the same set of genes.

In the search for evolutionary differences, comparative analysis of the developmental processes that take place in the pallium of the different groups of animals are being carried out. In mammals, neural progenitor cells sequentially produce multiple cell subtypes from the deep to the upper layers of the cortex in an inside-out order, so that later born neurons migrate radially past the deep layer neurons, and become the upper layers (Shen et al., 2006; reviewed in Florio and Huttner, 2014). This sequential production chronologically switches on and off the expression of fate determining transcription factors, which assign layer specific phenotypes to the differentiated neurons and eventually served as layer-specific neuronal markers (Molyneaux et al., 2007). However, in birds this inside-out pattern does not exist, but it has recently been proposed in chicken and turtle that single neural progenitor cells produce comparable neuron subtypes in the same chronological order as in mammals (Suzuki and Hirata, 2014). In addition, a correspondence was found between the genes expressed specifically in layer 5 and layer 2/3 and those expressed in the medial and lateral pallial domains of birds (Suzuki and Hirata, 2014), and a recapitulation of the mammalian inside-out arrangement was proposed (Suzuki et al., 2012; Suzuki and Hirata, 2013). These theories are being debated openly and intensely by authors strictly opposed, arguing that they leave out of consideration other parts of the pallium, making “pallium” and “cortex” equivalent, disregarding topologic developmental homology criteria in relation to the widely accepted tetrapartite pallium model, currently validated for all gnathostomes (see discussion in Puelles et al., 2016). In the case of *Xenopus*, based on the present assays, what we now know is that the developmental timing of the amphibian pallium, before and after the telencephalic evagination, does not follow this chronology and that the neurogenesis is randomly distributed. The PH3-ir cells are homogeneously distributed dorsoventrally and through the pallium itself, and this is in line with the results on BrdU labeling. In addition, in *Xenopus* the BrdU assays showed that neurons are generated in an outside-in order (the oldest neurons located to the pallial surface), as in the rest of non-mammalian vertebrates (present results, summarized in **Figure 11**).

In this context, the cells generated in each ventricular sector of the pallium migrate toward the mantle layer using the guide of the radial glial fibers, and each area that is formed in the pallium during development constitutes a radial unit. In *Xenopus*, RGc were observed using vimentin and BLBP as specific markers at late developmental stages (D’Amico et al., 2011) and the location of the labeled cells was used to define the vz (Coumailleau and Kah, 2014; Domínguez et al., 2015). The simultaneous observation of DAPI and BLBP allowed us to identify the radial organization in the pallium of *Xenopus*. As expected, the columns of nuclei observed with DAPI follow the arrangement of the radial glial fibers, showing a radial orientation with respect to the ventricular surface. However, it is not possible to discern different pallial subdivisions, because throughout the pallial surface this radial organization is comparable. Only in larvae from the period of metamorphosis is possible to recognize the VP within the telencepalic hemisphere, and corroborate the presence of higher cells numbers aggregated into nuclei, and this corresponds a slightly oblique orientation of the radial glia toward more ventral positions (present results), in line with the previously described migration routes described in the telencephalon of *Xenopus* (Moreno et al., 2008b).

### Analysis of Progenitor Cells

In the pallium of *Xenopus* at early developmental stages the expressions of Lhx2, Pax6, Sox2 and BLBP characterize primary precursor cells, which can be identified as RGc. They are mitotic cells located in the vz (summarized in **Figure 11**). The cells located separated from the vz that express Lhx2 or Pax6 (without co-expression), are not labeled with PH3 and show a postmitotic phenotipic appearance, in concordance with their anatomical position in regions described later in development and juvenile stages (Bandín et al., 2013, 2014).

At early stages, the Sox2-ir cells in the vz that co-express DCX and are mitotic (PH3+) would correspond to neuroblasts during the last part of the proliferation/migration/differentiation processes (reviewed in von Bohlen und Halbach, 2011). After stage 42, only part of the Sox2/DCX cells outside the vz are mitotic and likely correspond with different developing cells that would include IPs (the mitotic population), which in *Xenopus* do not express Lhx2 or Pax6 (present results). Until those embryonic stages, the animal is in an active neurogenic period but later, at the beginning of the larval stages, the situation changes, and neurogenesis is in a quiescence period (see **Figure 11**). From prometamorphosis, Lhx2 and Pax6ir expressing cells are present in the medial and VP, and some cells co-express both markers. The later population found at this highly active neurogenic moment could represent new IPs that have just abandoned the ventricle, since the number of double labeled cells is higher close to the vz and decreases toward the mantle.

In this context it was mandatory to analyze the expression of Tbr2 in the *Xenopus* developing pallium. Data about Tbr2 expression in the brain of *Xenopus* were previously obtained by *in situ* hybridization (Bachy et al., 2002; Moreno et al., 2003; Brox et al., 2004). Actually, the distribution of Tbr2 protein, localized by immunohistochemistry, and its mRNA in the brain of developing *Xenopus* seems to be highly coincident at early developmental stages (Moreno et al., 2003; present results). However, from the initial larval stages the level of expression and the capacity for detecting mRNA decay (Moreno et al., 2003). In the present analysis we observed that the Tbr2-ir cells separated from the vz do not express markers of progenitor cells and, more importantly, they are not mitotic cells. Based on our own results, it seems that there are IPs in *Xenopus*, but they do not express Tbr2.

In mammals, Pax6 is first expressed by vz precursor cells and, subsequently, an additional diffuse band of Pax6 cells appears in the svz, but the spatiotemporal sequence of appearance varies across species (Martínez-Cerdeño et al., 2012). Thus, in the rat and ferret most Pax6-expressing cells remain in the vz throughout neurogenesis (at the beginning of neurogenesis they represent about 90% of the vz cells), but when it is completed the Pax6 vz cells dropped rapidly (Martínez-Cerdeño et al., 2012). Additionally, in mammals the RGc intensely express Sox2, which it is only weakly expressed in IPs. In comparison, the marker of the RGc population Pax6 (Malatesta et al., 2000, 2003; Noctor et al., 2001; Anthony et al., 2004) is similarly expressed in the vz and svz, and most Sox2- positive cells within the vz also express Pax6, demonstrating that both transcription factors are highly expressed in RGc. Here we have demonstrated similar results for the case of *Xenopus*.

The comparison of the expression pattern of Sox2 and Pax6 with that of Tbr2, that labels the IPs (Bulfone et al., 1999; Kimura et al., 1999; Englund et al., 2005; Arnold et al., 2008; Sessa et al., 2008), it is highlighted that the Tbr2-positive IPs are primarily located in the svz and express weakly Sox2, but a subpopulation of the Tbr2-expressing cells that highly express Sox2 are located scattered throughout the vz/svz boundary and most likely represent newly generated IPs migrating out of the vz (Hutton and Pevny, 2011). Thus, in mammals Sox2 and Pax6 show low expression in the Tbr2+ IPs. It was also demonstrated the lack of Sox2 expression in the Tbr1-positive cells populating the developing marginal zone. The separate expression of Sox2 and Tbr1 indicates that Sox2 must be turned off before the cells reach the identity of Tbr1-positive neocortical neurons. In fact, it seems that Sox2 is irreversibly turned off in post-mitotic neurons close to their terminal differentiation (Bani-Yaghoub et al., 2006). Comparatively in *Xenopus*, Sox2-ir cells do not express any other marker of postmitotic cells in the svz, such as Tbr1, Lhx2, Pax6 or CR. However, recent gene profiling data highlighted the selective expression of Sox1 and Sox2 in cortical interneurons, in contrast to the lack of expression in cortical projection neurons (Liu et al., 2015), suggesting that it might be of importance for the specification of the interneurons, their maturation, and/or function. Again, at least in *Xenopus*, the pallial CR+ interneuron population (Morona and González, 2008) does not express Sox2 (present results).

In the case of the avian pallium, Pax6+ cells were located in the vz of the chicken and in individual cells superficial to the vz and svz, substantially separated from the Tbr2+ cell band. These Pax6 superficial cells do not express Tbr2 and are actively dividing cells, likely representing translocated RGc, thus opening the hypothesis of a conserved precursor cell type in birds (Martínez-Cerdeño et al., 2016). With respect to the IPs, Martínez-Cerdeño and cols. described that observable condensed chromatin indicates active division in some of the Tbr2+ cells in the chicken, as in mammals, postulating a conserved IPs phenotype in mammals and birds (Martínez-Cerdeño et al., 2016). Paradoxically, at the same time other authors following another line of thinking about the evolution of the IPs cells described abventricular mitosis in chickens, and Tbr2+ cells that did not display proliferative activity (Nomura et al., 2016). Based on that, it was proposed that in the developing avian telencephalon basal progenitors (IPs) are distinct from the Tbr2+ cells and they would represent different cell lineages (Nomura et al., 2016). They based their conclusions in the fact that they hardly ever found BrdU- labeled cells or EdU-labeled cells or mitotic cells expressing PH3+ among the population of Tbr2+ cells in the chicken, whereas most Tbr2+ cells were labeled with NeunN. It was concluded that theTbr2+ cells in the chicken do not display characteristics of cycling progenitors (Nomura et al., 2016). In addition, Tbr2 over expression in the chicken DP did not result in an increase of IPs (Nomura et al., 2016). Although they proposed possibilities about the origin of IPs in amniotes, Nomura et al. (2016) support that IPs could derive from a primitive population of mitotic cells separated from the ventricle, and that Tbr2+ cells acquired proliferative potential as IPs in placental mammalian lineages. In line with this discussion, analysis in metatherian mammals increase the complexity of the problem, since in this models it was proposed the existence of an aggregation of abventricular mitosis (Cheung et al., 2007), but significant numbers of Tbr2-expressing cells basal to the vz, which are not mitotic cells, have also been described Puzzolo and Mallamaci (2010).

In the turtle, Tbr2 expression was found in cells located in the ventricular lining and at the surface of the ventricle and throughout the svz and some of these cells were mitotic cells (Clinton et al., 2014; Martínez-Cerdeño et al., 2016), resembling the situation of mammals. But the organization of the turtle svz is much more simpler than that described in chickens, which is closer to mammals (Martínez-Cerdeño et al., 2016). Additionally, in the turtle numerous Tbr2+ cells are located during neurogenic periods in the proliferative zone of the dorsal ventricular ridge (DVR) and scattered Tbr2+ cells are also distributed throughout the dorsal cortex in the vz, resembling a embryonic rat cortex before the formation of the svz, while in the DVR the situation resembles to a proper svz (Clinton et al., 2014). It has been discussed that this could support the hypothesis about the evolutionary similarities between the sauropsid DVR and the mammalian dorsal cortex (Dugas-Ford et al., 2012; Martínez-Cerdeño et al., 2016). However, the DVR is a derivative of the VP, which precludes this comparison (Puelles et al., 2000). The fact that the turtle DVR has a svz much more organized than the dorsal cortex might be related to the different strategies to achieve encephalization based on pallial expansion and, thus, increasing the volume of specific pallial regions, such as the neocortex of mammals versus the DVR of sauropsids. The existence of the IPs in the svz would be directly implicated in the development of the complexity of the particular cortical structure and, thus, the adult turtle dorsal cortex is simpler than the DVR derivatives.

The situation in lizards differs significantly from that of turtles because abventricular mitotic cells are not detected (Martínez-Cerdeño et al., 2016). The vz harbors Pax6+ cells but also Tbr2+ cells, and an organized subventricular band superficial to the vz is not formed, opening the hypothesis of the absence of IPs in lizards. Actually, in several previous studies in different reptilian species, including crocodiles and geckos, cells that might be identified as pallial basal mitotic cells during embryogenesis were not detected (Charvet and Striedter, 2011; Nomura et al., 2013b). Therefore, IPs may have evolved independently in turtles and/or these IPs may have been present in ancestral vertebrates and have been lost in several sauropsid groups. Interestingly in evolutionary terms, it has recently been demonstrated that turtles are closer relatives of crocodiles and birds than of lizards (Crawford et al., 2014). From all studies to date it becomes clear that Tbr2+ cells were already present in the pallium of the common ancestor of both reptiles and mammals, thus likely in all amniotes, but their capabilities in mammals to form a band superficial to the vz that will give rise to the svz is what varies among other groups (Noctor et al., 2008).

**FIGURE 12:**
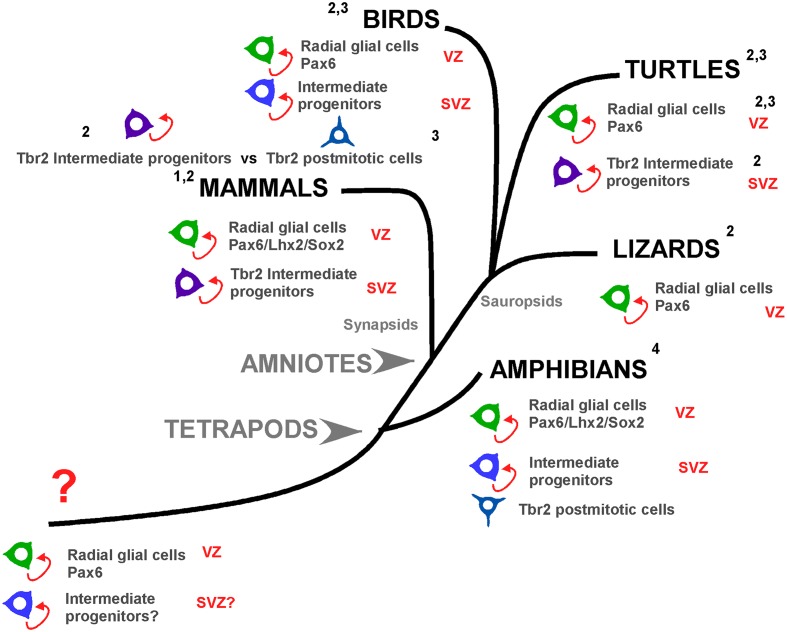
**Pallial progenitor cells subtypes during evolution**. Cladogram showing the pallial progenitor cells subtypes present in the different groups of tetrapod vertebrates. In mammals, the apical progenitors, or radial glial cells, proliferate and generate basal progenitors, or intermediate progenitors, which include Tbr2+ cells and specially the distinct svz, defined on the basis of the distribution of Tbr2+ cells. In birds, apical progenitors exist but there is a controversy about the nature of the basal progenitors. Nomura and collaborators (3) propose that basal progenitors and Tbr2+ cells are different cell populations. In contrast, Martínez-Cerdeño and collaborators (2) propose that birds posses a distinct svz with intermediate progenitors based on Tbr2+ expression. In lizards Tbr2 cells are present but there are not abventricular mitotic cells. In turtles there are Tbr2+ dividing cells, but a svz-like structure is only defined in the dorsal ventricular zone. Finally, in *Xenopus* the apical progenitors are conserved and there are abventricular mitosis, but the Tbr2+ cells are not dividing cells (present results). Thus, in evolutionary terms, the present evidences show that before the amniote evolution, in the tetrapod common ancestor subapical mitotic cells appeared and those are the origin of the basal progenitors and possible a distinct svz. 1: Martínez-Cerdeño et al., 2012; 2: Martínez-Cerdeño et al., 2016; 3: Nomura et al., 2016, 4: present results.

In an attempt to get insight into the evolution of progenitor cell types in the pallium of vertebrates, the situation in anamniotes provides important new clues (see **Figure 12**). In the case of *Xenopus* it is evident the existence of abventricular mitotic cells (Nomura et al., 2016; present results). The Pax6+ cells observed in the vz are actively mitotic but those detected separated from the ventricle represent a postmitotic cell population that can be found until the adult (present results; Bandín et al., 2013, 2014). Cells expressing Tbr2 are present outside the vz but they are not mitotic cells (present results), whereas among the demonstrated Sox2/DCX expressing cells some are mitotic cells. Therefore, on the basis of the current results in *Xenopus* it seems plausible that in anamniotes IPs exist in the pallium but the signification of the Tbr2+ cells is still unknown, as it is unknown whether these cells are generated from RGc (apical progenitors) that upon leaving the vz acquire postmitotic characteristics. A small population of IPs is present in *Xenopus*, but in these basal progenitors at least part of the machinery is absent, or changed through the evolution of tetrapods.

## Author Contributions

Both authors had full access to all the data in the study and take responsibility for the integrity of the data and the accuracy of the data analysis. AG and NM devised the study. NM performed the experiments and the two authors contributed to the data analysis. NM led the figure preparation and wrote the majority of the article, further completed and edited by AG. Both authors approved the article.

## Conflict of Interest Statement

The authors declare that the research was conducted in the absence of any commercial or financial relationships that could be construed as a potential conflict of interest.

## Footnotes

1 https://imagej.nih.gov/ij/

## Abbreviations

Acc: accumbens
AOB: accessory olfactory bulb
DM: dorsomedial pallium
DP: dorsal pallium
CeA: central amygdala
LP: lateral pallium
MOB: main olfactory bulb
MP: medial pallium
OB: olfactory bulb
Pa: pallium
S: septum
SPa: subpallium
Str: striatum
VL: ventrolateral pallium
VP: ventral pallium
vz: ventricular zone
